# First Principles of Medicine

**Published:** 1838-01

**Authors:** 


### Art. XVIII.

*First Principles of Medicine.* By Archibald Billing, m.d. a.m., Member of the Senate of the University of London, &c. &c. *Second Edition.—London,* 1837. 8vo. pp. 130.

An idea, which must have been at some time or other entertained by every student accustomed to reflect on what he heard and saw, that, namely, of its being possible, out of the mass of ill-explained practical facts in medicine, to draw a clear continuous thread of medical principles, seems to have been the origin of this little work. The author did not allow his dissatisfaction with conventional explanations to exhale in general accusations of physic and physicians; but set to work to remove, if possible, the causes of what dissatisfied him.

"I visited," he says, "the different schools; and, though the students of each hinted, if they did not assert, that the other sects killed their patients, I found that, provided the physician of each was a man of talent and experience, the mortality was fairly balanced. I thought there must exist some true general principles by which the apparent inconsistencies of correct practice might be reconciled, and by which the contest between such systems as were essentially at variance might be decided. But, though there were to be found innumerable volumes of cases, and interminable heaps of insulated precedents, there was no treatise upon first principles. After twenty years of intense application to clinical practice, as a student, assistant, and professor, I ventured to publish what I had drawn up, condensed to 130 pages." (*Preface*, p. vi.)

We can imagine no more legitimate mode of producing a useful work than this; and Dr. Billing's opportunities of acquiring experience at the London Hospital, combined with the disposition manifested in these expressions, entitle his observations to great attention. Nor are we unmindful of the debt of gratitude owing to Dr. Billing for being one of the first, if not the very first, to practise *clinical* teaching, in the true sense of the word, in the London school, so early as 1822.

We meet in Dr. Billing, as in Dr. Latham, a witness of the negligence of the London students respecting the uses of the stethoscope; but those who were immoveable by higher motives may, perhaps, be startled into studying it by Dr. Billing's admirably directed remark, that many affectionate parents "will soon begin the very simple process of applying their ear to the chest, and thus put the medical attendant to shame."

Every teacher of medicine, passing yearly in review the principal phenomena of disease, must find, after lecturing some years, that he becomes master, by the sole power of reflection, of some of the facts which during that time physiology and minute anatomy, advancing with slow but continual pace, have succeeded in developing or establishing. That Dr. Billing, therefore, an active as well as independent thinker, should find materials for half-a-dozen pages of prefatory reclamations, does in no degree so much surprise us, as the good humour, little characteristic of reclamators, with which they are penned. Yet they comprehend important matters: the functions of nerves, the reflex principle, the theory of inflammation, the sounds of the heart, and the cholera; concerning all of which matters there has been much ink-shed. Thus, mortal men, using the intellect which God has given to them, make ever slow advances along the boundaries of knowledge, sometimes in column, and sometimes in equal line of march; and happy is it when, on lighting on some undiscovered truth, they do not turn round or aside, and contend with their followers or fellows, as is the wont of carnivorous creatures over their precarious food, disgracing the understanding and mocking the dignity of the human creature by the passions of the lower tribes of animals. It was a saying of Sir James Mackintosh, that, when asked in another world what he had been doing all his life-time in this, he should have no better answer to make than that "he had been talking." Of how many a physiologist the reply must be, "I have been fighting:" not from the calmness and silence of the study, with unwrinkled brow advancing, but with flushed cheek and soul-marks of intellectual blows.

Dr. Billing introduces the subject of the First Principles of Medicine by a brief survey of the leading facts in physiology. In speaking of the function of repair, he lays especial stress on inflammation, being the *notice* that repair is wanting, and also the process by which the spaces are prepared where the reparation (in a bone) is to take place, prepared by distention of the vessels, by sponginess induced in the part requiring repair; so that, if not stagnation, a sufficient retardation of the blood takes place "to allow of crystallization of bony matter." The use of the term inflammation will appear objectionable, perhaps, to many pathologists, who would admit that a peculiar condition of the part, accompanied by or passing on to inflammation, was the agent in giving the required notice. The state is probably a compound state, to be resolved into certain conditions of the nerves as well as of the blood-vessels.

Indeed, in other passages, Dr. Billing very distinctly explains that inflammation begins in the nerves, on which the capillaries depend for tone and energy. (P. 27.) In a subsequent page, (p. 35,) he seems to restrict this to *morbid* inflammation, in contradistinction to *healthy* inflammation. All this proves that our language is deficient in exact terms to express several morbid states of the blood-vessels, as well as of the nerves. It is not consonant with the impressions on the ordinary senses to admit, what Dr. Billing and others strenuously maintain, that in inflammation there is only diminished action of the arteries; that inflammation and congestion are but degrees of the same condition, &c. (P. 24.) The state of the arteries or of the capillaries, during inflammation, is not necessarily that of simple diminished action, *because* it is not a state of increased action. There is yet something undetected, and which determines the alterations of tissue that characterize inflammation.

Dr. B. maintains the elasticity of the arteries, and the non-vascularity of the circular fibres of the middle coat, which, allowing of separation laterally, and resisting dilatation, accommodate themselves to the elongation of the artery; in consequence of which the arteries are increased in capacity at each contraction of the heart, but by longitudinal stretching; whence arises the serpentine motion visible where the arteries are at all loose. He does not admit the active power of dilatation which some physiologists ascribe to the heart: and he considers the right auricle to be filled by a constant pressure and an equable stream, not by suction, or any effect of vacuum and atmospheric pressure; observing, that there can be " no suction (no atmospheric pressure) during natural respiration, for the glottis is sufficient to admit a free current of air; it is only in croup or laboured respiration of any kind that there can be any effect of atmospheric pressure." (P. 12.) The capability of the heart to send blood all over the frame is, he thinks, intelligible by a reference to the hydrostatic principle of fluid in bent tubes finding its level.

Dr. Billing's theory of animal heat would seem to be identical with the celebrated one of Dr. Crawford. He expresses it somewhat oddly; and the paragraphs, it will be seen, are oddly printed. (We retain his types.)

"The ANIMAL HEAT has been accounted for in different ways by several ingenious physiologists; from the aggregate of their opinions and experiments, I deduce, that *heat* is *extricated all over the frame*, in the *capillaries*, by the *action* of the *nerves*, during the *change* of the *blood*, from *scarlet* arterial to *purple* venous; and *also* whilst it is changing *in the lungs* from *purple* to *scarlet*.

"There is a perpetual *deposition*, by the capillary system, of *new matter* and decomposition of the *old* all over the frame, influenced by the nerves; in this decomposition there is a continual disengagement of carbon, which mixes with the blood returning to the heart, at the time it changes from scarlet to purple; this *decomposition* being effected by the *electric agency* of the nerves, produces *constant extrication of caloric;* again, in the lungs that *carbon is thrown off* and *united* with *oxigen*, during which *caloric* is again *set free;* so that we have in the LUNGS a CHARCOAL FIRE constantly burning, and in the OTHER PARTS A WOOD FIRE, the one producing *carbonic acid gas, the other carbon;* the *food supplying* through the circulation the *vegetable* (or what answers the same end, animal) *fuel*, from which the *charcoal* is *prepared* which is *burned* in the *lungs*.

"Thus the animal heat is kept up, the EVAPORATION of PERSPIRATION keeps the SURFACE COOL; but in inflammatory fevers where this is *deficient*, the body gets too *hot;* and in low fevers where the nervous influence is too low to keep up the full fire, the surface gets cooler than the natural standard." (P. 18.)

The phenomena of determination, blushing, &c. are explained on the principle, so strongly enforced by Dr. Parry, of diminished tonicity. Diminished secretions, coexistent with increased quantity of blood in the secreting organ, are explained by the readier passage of the arterial blood through the enlarged capillary vessels to the veins, although with slower current, without obstruction, spasm, or error loci; a diminished supply being the consequence in the capillaries of which it is the office to secrete. This applies to that form of diminished secretion in which the heart retains its usual power; and the relaxation of the capillaries is ascribed to a want of nervous energy. Secretion may also be diminished from a failure in the injecting power.

In treating of the means of allaying inflammation and removing its consequences by means auxiliary to bleeding, Dr. Billing refers to the theory of capillary distension, with applications that may appear novel to some of his readers; as, for instance, when speaking of this use of mercury:

"I believe that mercury removes morbid growths by starving them, by contracting the capillaries, and not by increasing absorption, as is a commonly received opinion: it may be said that the swelling of the gums and fauces from mercury is a contradiction of their being starved; but, though swelled, they are worse nourished, are spongy, weak, soft; as a limb may be swelled by dropsy, though actually emaciated as to its natural substance: another apparent objection to this idea of starvation is that mercury stops ulceration; but in many of our medical explanations we appear to 'blow hot and cold;' it stops ulceration, for the same reason in one case that it produces it in another; it contracts the capillaries; so that a healthy part is ulcerated by what contracts its nutrient capillaries *from* a natural state, an unhealthy ulcer is stopped by what contracts its relaxed capillaries *to* a natural state." (P. 39.)

In this quotation we have been obliged to take considerable liberties with the punctuation, which has evidently been quite overlooked in the correction of the press in Dr. Billing's book, and is so imperfect and capricious in every page as to confound and obscure the meaning, particularly to those to whom the subjects treated of are not familiar. This may seem a slight fault for a critic to notice, but it would be absolutely better to print a book without any stops at all than to point it with commas and semicolons thrown in at random, and constituting not stops, but hinderances. Such blemishes are always more to be regretted in porportion to the excellence of the publication, and are now so rare in works of respectability that their presence is seriously detrimental to a writer's reputation. We find no printer's name in any part of the book, and the author's time may be better engaged than in correcting proofs, but surely Mr. Highley should have some regard to sheets which issue from his shop, and some regard for those who publish with him.

An attempt is made to clear away the obscurity attached to the terms stimulant and sedative, as employed by many writers, and partly arising from the hypothetical notion that all sedatives became such in consequence of a primary stimulant operation.

"Stimulants produce stupor or confusion of ideas by overpowering the brain with arterial blood, from the *increased action* of the *heart* which they occasion; for, though arterial blood is the source from which the capillaries of the blood prepare or secrete the nervous influence, *over-injection* diminishes secretion (as in the kidneys, &c.); but mere increased action of the heart is not sufficient to produce the over-injection, the *stimulant* itself being circulated to the brain at the same time, *expends* by its action on it, the *nervous influence* which it meets with there; besides that, it interferes with the generation of more, so that the capillaries, from the exhaustion of nervous influence, became more *distensible:* the proximate cause, then, of *coma* from *stimulants* is *congestion*, or *plethora.*

"Sedatives, on the contrary, *diminish* the *injection* of the brain, at the same time *repressing* the *nervous influence;* so that the proximate cause of *coma* from *sedatives* is *inanition.*

"This will explain one cause of the confusion of terms: sedatives are sometimes miscalled stimulants when they relieve the coma of stimulants, or the drowsiness of fatigue, or other plethora, because this relief is called rousing or awakening, as by tobacco-snuff, digitalis, green tea. A stimulant, wine, given to a person fatigued produces sleep.

"By the term *sedative* is *not* to be understood a *putting to sleep,* but whatever has for a time the effect upon the nervous system as if it had been refreshed by sleep; for by checking the expenditure of nervous influence in the organs, there is more of it left at the disposal of the brain, but this of course has not the restorative effect of sleep (which does not diminish the supply of arterial blood to the brain by checking the action of the heart); on the contrary, though the ideas are rendered free at first, exhaustion will at length produce the coma of inanition." (P. 47.)

Dr. Billing considers the proximate cause of fever to be an "injured state of the brain and nerves," and to this injury he gives the name of inflammation. He acknowledges that we do not always see proofs of this after death, but ascribes this circumstance to the nature of the nervous tissue. "This injury of the nerves (fever) may," he says, "be produced either by disease of some part in the body, as traced above, or by a poisonous matter introduced from without in the form of effluvia, the effect of which poison is to debilitate the nervous system, and consequently all other parts as depending upon it for energy." (P. 92.) Of this kind of theory and language we have already had to express our dissatisfaction. Dr. Billing's remarks on the treatment of fevers are, however, indicative of much experience and sound judgment; and his observations on the neuroses contain ingenious and interesting matter for reflection. But we must accuse him of being the worst *book-maker* that we ever met with. No table of contents, no division into chapters or sections, no title-pages, and no index, guide the reader through what thus appear desultory and unconnected pages. Still another edition is required, in which these things may be corrected, the printing attended to, and something more of form given to the whole work.

